# Incidence, causes and phenotypes of acute seizures in Kenyan children post the malaria-decline period

**DOI:** 10.1186/s12883-015-0444-8

**Published:** 2015-10-06

**Authors:** George K. Serem, Charles RJC Newton, Symon M. Kariuki

**Affiliations:** KEMRI-Wellcome Trust Research Programme, PO Box 230, Kilifi, Kenya; Department of Psychiatry, University of Oxford, Oxford, UK; Nuffield Department of Medicine, University of Oxford, Oxford, UK

**Keywords:** Acute seizures, Causes, Falciparum malaria, Incidence, Phenotypes

## Abstract

**Background:**

Acute seizures are a common cause of paediatric admissions to hospitals in Africa, and malaria is an important cause of seizures in endemic areas. Malaria has declined in the past decade whilst neonatal admissions have increased, both which may affect the incidence and phenotypes of acute seizures in African children.

**Methods:**

We examined the effect of recent decline in malaria and the increasing burden of neonatal admissions on the incidence, causes and phenotypes of acute seizures admitted to hospital from 2009–2013. We used logistic regression to measure associations and Poisson regression to calculate the incidence and rate ratios.

**Results:**

The overall incidence of acute seizures over the 5-year period was 312 per 100,000/year (95 % CI, 295–329): 116 per 100,000/year (95 % CI, 106–127) for complex seizures and 443 per 100,000 live births (95 % CI, 383–512) for neonatal seizures. Over the period, there was an increase in incidence of seizures-attributable to malaria (SAM) (incidence rate ratio (IRR) = 1.25; *p* < 0.001), but neither non-SAM (IRR = 1.03; *p* = 0.569) nor neonatal seizures (IRR = 0.99; *p* = 0.905). Important causes of acute seizures were malaria (33 %) and respiratory tract infections (19 %); and for neonatal seizures were neonatal sepsis (51 %), hypoglycemia (41 %) and hypoxic-ischemic encephalopathy (21 %). Mortality occurred in 6 % of all acute seizures, being more common in complex seizures (8 %) and neonatal seizures (10 %) than other seizures (*p* < 0.001 for both comparisons).

**Conclusions:**

Acute seizures remain common in children despite a decline in the incidence of malaria; suggesting that causes for these seizures need to be prevented in the community.

## Background

Acute seizures are common in children admitted to African hospitals [1] and malaria is the most important cause of seizures in endemic areas [2]. The proportion of complex seizures (repetitive, prolonged and/or focal) in malaria-endemic area is twice that reported in other settings, where seizures with fever have been studied [3, 4]. In endemic areas, seizures in children are associated with acute febrile infections such as malaria, respiratory tract infections and gastroenteritis [1].

The incidence of acute seizures in Kilifi, Kenya (a malaria endemic area), before the decline in malaria was high (425-650/100,000/year) [1, 2], and most complex seizure phenotypes were attributable to malaria [2]. We hypothesised that the incidence of acute seizures and the proportion with complex phenotypes depend on changes in transmission intensity of malaria. From early 2000–2008, admissions with malaria and seizures declined [2, 5], and so was the associated mortality [6]. In addition, neonatal admissions have increased, suggesting emergence of other causes of seizures during the neonatal period or improvement in hospital access in rural hospitals [7]. These changes may affect the incidence, causes and phenotypes of seizures in children admitted to hospital.

We examined the effect of the recent widely documented decline in malaria and the increasing burden of neonatal admissions on the incidence of acute seizures, the prevalence of causes and phenotypes. We examined children admitted to a rural county hospital between 2009 and 2013, which are post malaria decline years.

## Methods

### Study site and population

This study was conducted in Kilifi Health and Demographic Surveillance System which is situated on the Kenyan coast about 50 kilometres from Mombasa and has a population of about 270,000. The population is comprised of a Mijikenda speaking community, who are mainly subsistence farmers and a few fishermen. The transmission intensity of malaria has reduced this area, resulting in reduction in morbidity and mortality due to malaria [5], in particular seizures-attributable to malaria (SAM) [2].

### Admissions and management

A total sample of 3074 children aged 13 years and below with a history of seizure(s) during that period of the presenting illness were admitted to Kilifi County Hospital (KCH) from the year 2009–2013. Children with severe malaria were treated with first-line intravascular artesunate [8], with broad-spectrum antibiotics. Children admitted with seizures lasting 5 minutes were first treated with intravenous diazepam, and for those whose seizures did not stop within 10 minutes a second dose of diazepam or intramuscular paraldehyde was given [9]. Seizures lasting 15–20 minutes were managed with phenobarbital, and those continuing further with phenytoin. Seizures refractory to phenytoin were managed in consultation with senior clinicians, who would often recommend another dose of phenobarbital or midazolam since artificial ventilation was not available.

### Review of case notes and data extraction

Clinical files for these children were reviewed to determine which admissions had acute seizures. Data for 1063 records was obtained from reviewing the patient’s case notes, for the remaining 2011 records data was obtained from the hospital inpatient database. Case notes were independently reviewed by Serem KG (who examined all case notes) and Kariuki SM (who randomly reviewed half of the case notes); with any differences in phenotypes resolved through consensus. The data extracted includes among others: the age in months of the patient, diagnosis, seizure type, medications received and blood workups. Data were extracted into Excel spread sheet, before being transferred into the analysis software.

### Definitions of terms

Phenotypes of seizures are defined in Table 1, and were based on ILAE recommendations [10–12], that have been validated in previous studies in this area [13, 14]. Neonates were defined as those aged 28 days or younger [7]. Malnutrition was defined as a weight for age z-scores (WAZ) < −2 or mid-upper arm circumference (MUAC) <11 centimetres or a clinical diagnosis on discharge. Meningitis was defined as cerebrospinal white cell count >50/μL [15]. Anaemia defined as a haemoglobin concentration <50 g/L, thrombocytopenia as a platelet count <150,000/μL, hypoglycemia as glucose concentration of <3 Mmol/L, hyponatremia as sodium concentration of <125 Mmol/L, and hyperkalemia as potassium concentration of >4.5 Mmol/L.

**Table 1:** Definition of phenotypes of acute seizures

Phenotypes of seizures	Definition
Acute seizures	These are seizures that are associated with an acute event such as a febrile illness, acute haemorrhagic stroke, poisoning or electrolyte imbalance.
Acute symptomatic seizures	Acute seizures associated with neurological causes e.g. seizures due to malaria or bacterial meningitis.
Febrile seizures according to ILAE	Seizures associated with fever (temperature ≥38.5 °C) that occur in a sick child aged between a month to 6 years old, and the cause of fever does not involve the brain.
Febrile seizures according NIH	Seizures with fever usually occurring in child aged between 3 months to 6 years in absence of neurological involvement.
Overall definition of febrile seizures	Seizures with fever (temperature ≥38.5 °C) that occur in 2-8 % of children aged between 1 month to 6 years and exclude acute symptomatic seizures.
Definite prolonged seizures	Seizures that last up to ≥10 minutes, and timing is witnessed by a clinician or nurse.
Probable prolonged seizures	Seizures which did not stop after first dose of diazepam or continuing seizures that required second/third line antiepileptic drugs (phenobarbital/phenytoin).
Possible prolonged seizures	These are prolonged seizures based on parental history i.e. history of a seizures lasting ≥10 minutes, based on parents or caretakers reported.
	A history of a child convulsing all the way to hospital for those living at least half a kilometre away.
Overall definition of status epilepticus	Seizures that last ≥30 minutes accompanied by loss of consciousness or intermitted seizures over a period of ≥30 minutes without regaining consciousness in between the seizures.
Definite status epilepticus	Seizures fulfilling the definition of status epilepticus as above, but were witnessed/ observed by a clinician or a nurse and the timing is well documented in the child’s clinical notes.
Probable status epilepticus	Prolonged or continuing seizures which required the administration of AEDs such as phenobarbital or phenytoin.
Possible status epilepticus	A parental/caretakers history of seizures lasting ≥30 min, when the child was not convulsing on admission.
	A parental/caretakers’ history of child convulsing all the way to hospital for those living at least a kilometre away.
Focal/partial seizures	Seizures localised to or involving one body part e.g. twitching of one side of the facial structures, cycling movements of one limb, Todd’s paresis of one body side and motor deficits involving one side of the body post-ictally.
Generalised seizures	Seizures involving the entire body parts.
Repetitive seizures	Two or more seizures in the current illness or within 24 hours.
Complex seizures	Seizures that have focal, repetitive and/or prolonged (including status epilepticus) features.

### Statistical analysis

The data were analysed using the STATA 13 statistical software (Stata Corporation, Texas, USA). The association between complex seizures and social demographic characteristics and other clinical variables was measured using logistic regression, and variables with a p-value <0.25 were used to build a multivariate model in a stepwise regression technique. Hosmer-Lemeshow test was used to test the goodness of fit in logistic regression model. For comparison of continuous variables, the Student t-test was used and if data did not have a Gaussian distribution, Mann–Whitney U-test was used. For comparison of categorical variables, the Pearson’s Chi-square test or Fisher’s exact (where observations were infrequent) were used. Proximate causes of acute seizures mentioned in the literature were reported here as percentages of occurrence among admissions.

For different phenotypes of acute seizures, we computed overall incidence, then stratified incidence by year, age group and falciparum malaria status. Incidence was computed by dividing number of children with acute seizures by person-years of observation for all admissions and by live births for neonatal admissions. The trend of incidence of acute seizures was determined using the Poisson regression technique. The trend for all admissions was repeated for neonatal seizures, non-malarial seizures, malarial seizures, and malarial seizures with a parasite density ≥2500 μ/L, a sensitive and specific cut-off for defining seizures attributable to malaria that was modelled with logistic regression techniques in previous studies [2, 16].

### Ethics, consent and permissions

This study was approved by the Kenyan Ethical Review Committee (SSC No 2599) and parents of children participating in the study gave a written informed consent.

### Data availability

These data are stored securely within the KEMRI-Wellcome Research Programme servers as was agreed with participants during consenting and can be availed upon formal request.

## Results

### General description

**Fig. 1:**
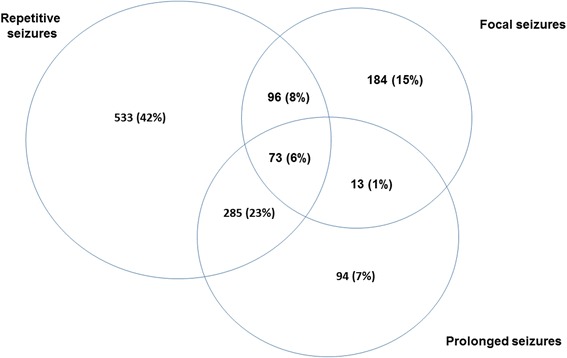
Overlap of phenotypes of seizures over the study period (*N* = 1263). There was substantial overlap of the three phenotypes of complex seizures namely prolonged, repetitive and/or focal seizures

Over the 5 year period, 2742 children with acute seizures were admitted to KCH, of whom 1567 (57 %) were males (Table 2) and 182 (7 %) had neonatal seizures. Nearly half (46 %) of children were admitted with complex seizures, and different phenotypes of complex seizures showed significant overlap with each other (Fig. 1). Malaria parasitemia was detected in 38 % of all admissions and in 36 % of complex seizures (36 % of prolonged seizures, 39 % of convulsive status epilepticus, 31 % of repetitive seizures and 22 % of focal seizures). Malnutrition was present in 23 % of all admissions, and was more frequent in children with complex seizures (27 % vs. 21 %; *p* < 0.001), but less frequent in those with malaria parasitemia (16 % vs. 27 %; *p* < 0.001). Complex seizures were independently associated with mean corpuscular volume in a multivariable logistic regression model (odds ratio = 0.92 (95 % CI, 0.85-0.99); *p* = 0.033).

**Table 2:** Distribution of social demographic, laboratory and clinical characteristics among children with seizures

Variable	Complex seizure (*N* = 1260)	Simple seizure (*N* = 1482)	Total (*N* = 2742)	Odds ratio (95 % CI)	p-value
Age: median (interquartile range (IQR))	28.4 (12.2-51.9)	27.9 (15.8-44.4)	28.1 (14.5-47.5)	1.00 (1.00-1.00)	0.171
Sex (male)	711/1263 (56 %)	856/1486 (58 %)	1567/2749 (57 %)	0.95 (0.82-1.10)	0.489
Clinical signs and symptoms					
Median respiratory rate (IQR)	40 (32–50)	38 (30–47)	38 (32–48)	1.02 (1.01-1.02)	<0.001
Median heart rate (IQR)	149 (128–166)	152 (134–167)	150 (132–167)	1.00 (0.99-1.00)	0.003
WAZ^a^	−2 (−2 - -1)	−1 (−2 - 0)	−1 (−2 - -1)	0.97 (0.92-1.03)	0.379
MUAC^b^	14.0 (13.0-1.05)	14.5 (13.5-15.2)	14.2 (13.0-15.0)	0.92 (0.89-0.96)	<0.001
Prostration	513 (41 %)	293 (20 %)	806 (29 %)	2.67 (2.27-3.13)	<0.001
Median temperature °C (IQR)	38.0 (37.0-39.0)	38.3 (37.4-39.2)	38.2 (37.2-39.1)	0.86 (0.81-0.91)	<0.001
Temperature gradient	90/1261 (7 %)	86/1470 (6 %)	176/2731 (6 %)	1.24 (0.91-1.68)	0.173
Laboratory investigations					
Median Hb levels (IQR)	10.0 (8.6-11.2)	10.1 (9.1-11.1)	10.2 (8.9-11.1)	1.02 (0.98-1.05)	0.379
Median glucose levels (IQR)	4.8 (3.8-6.1)	4.7 (3.9-5.8)	4.7 (3.8-5.9)	1.02 (0.99-1.06)	0.169
Median sodium levels (IQR)	134 (130–137)	134 (132–137)	134 (131–137)	0.99 (0.96-1.00)	0.016
Median potassium levels (IQR)	3.9 (3.5-4.3)	3.8 (3.4-4.2)	3.8 (3.4-4.2)	1.21 (1.06-1.39)	0.005
Median BCS (IQR)	4 (2–5)	5 (4–5)	5 (4–5)	0.71 (0.69-0.76)	<0.001
Median hematocrit (IQR)	31.3 (27.0-34.6)	31.5 (28.5-34.1)	31.4 (27.9-34.3)	1.00 (0.99-1.02)	0.375
Median blood oxygen saturation % (IQR)	98 (95–100)	99 (97–100)	98 (96–100)	0.96 (0.95-0.97)	<0.001
Median mean corpuscular volume (IQR)	73.4 (66.0-81.0)	72.0 (65.0-78.0)	73.0 (65.0-79.0)	1.02 (1.01-1.03)	<0.001
Median platelets (IQR)	292.0 (146.5-421.0)	314.0 (179.0-433.0)	306.0 (165.0-427.0)	1.00 (1.00-1.00)	0.032
Parasite density ≥2.5x10^3^/μL	318/1263 (25 %)	344/1486 (23 %)	622/2749 (39 %)	1.12 (0.94-1.33)	0.215
Median parasite density (IQR)	2976 (0–165880)	0 (0–50640)	0 (0–101710)	1.00 (1.00-1.00)	0.001
Slide positive for malaria	377/1060 (36 %)	383/1332 (29 %)	760/2392 (38 %)	1.37 (1.15-1.63)	<0.001
Creatinine	45 (37–58)	50 (38–64)	47 (37–60)	1.00 (1.00-1.00)	0.382

^a^WAZ = weight for age; ^b^MUAC = mid-upper arm circumference BCS = Blantyre coma score; CI = confidence interval; IQR = interquartile range

### Possible causes of acute seizures

The most common causes of the 2742 admissions with acute seizures were malaria (n = 899, 33 %)), respiratory tract infection (n = 532, 19 %)), fever of unknown origin (n = 467, 17 %)) and hypoglycemia (n = 274, 10 %). Other less common causes of acute seizures were as shown in Table 3. Malaria was an important co-morbidity in those with malnutrition (23 %).

**Table 3:** Possible causes of acute seizures

Possible cause	Complex seizure *n* = 1263 (%)	Simple seizure *n* = 1486 (%)	Overall *n* = 2742 (%)	p-value
Malaria	477 (38 %)	422 (28 %)	899 (33 %)	<0.001
Respiratory tract infections	180 (14 %)	352 (24 %)	532 (19 %)	<0.001
Fever of unknown origin	161 (13 %)	306 (21 %)	467 (17 %)	<0.001
Hypoglycemia	104/832 (13 %)	103/1184 (9)	207/2016 (10 %)	0.006
Gastroenteritis	56 (4 %)	95 (6 %)	151 (5 %)	0.025
Hyponatremia (<125Mmol/L)	50/759 (7 %)	31 (3 %)	81 (5 %)	0.003
Meningitis	69 5 %)	58 (4 %)	127 (5 %)	0.052
Neonatal sepsis	72 (6 %)	39 (3 %)	111 (4 %)	<0.001
Unknown encephalopathy	73 (6)	31 (2 %)	104 (4 %)	<0.001
Low hemoglobin (<5 g/L)	35/1047 (3 %)	34/1338 (3 %)	69/2385 (3 %)	0.246
Urinary tract infection	16 (1 %)	25 (2)	41 (1 %)	0.370
Hypoxic-ischemic encephalopathy (birth asphyxia)	30 (2 %)	9 (1 %)	39 (1 %)	<0.001
Trauma	16 (1 %)	18 (1 %)	34 (1 %)	0.896
Skin disease	11(1 %)	19 (1 %)	30 (1 %)	0.305
Septicemia	12 (1 %)	15 (1 %)	27 (0 %)	0.875
Anemia	13 (1 %)	5 (<1 %)	18 (1 %)	0.025
Sickle cell disease	8 (1 %)	7 (<1 %)	15 (1 %)	0.565
Undetermined	5 (<1 %)	5 (<1 %)	10 (<1 %)	0.797
Ear infection	2 (<1 %)	5 (<1 %)	7 (<1 %)	0.356
Immunosuppression (HIV exposed)	2 (<1 %)	4 (<1 %)	6 (<1 %)	0.535
Eye infections	1 (<1 %)	4 (<1 %)	5 (<1 %)	0.244
Poisoning	2 (<1 %)	0 (0 %)	2 (<1 %)	0.125
Prematurity	2 (<1 %)	0 (0 %)	2 (<1 %)	0.150
Other causes	12 (0.95 %)	15 (1.01 %)	27 (0.98 %)	0.875
Outcome: died	104 (8 %)	54 (4 %)	158 (6 %)	<0.001

A number of several possible causes were significantly more frequent in complex seizures compared to simple seizures: malaria (*p* < 0.001), respiratory tract infections (*p* < 0.001), fever of unknown origin (*p* < 0.001), encephalopathy of undetermined cause (*p* < 0.001), neonatal sepsis (*p* < 0.001), hypoglycemia (*p* = 0.006) and hyponatremia (*p* = 0.003). On the other hand, gastroenteritis was more common in simple than complex seizures (*p* = 0.025). Hypoglycemia was significantly more common in those with neonatal seizures compared to those without (54/133 (41 %) vs. 153/1881 (8 %); *p* < 0.001) and as was sepsis (95/182 (52 %) vs. 16/2565 (1 %) *p* < 0.001) and hypoxic-ischemic encephalopathy (39/182 (21 %) vs. 0/2565 (0 %); *p* < 0.001).

### Overall Incidence of acute seizures

**Fig. 2:**
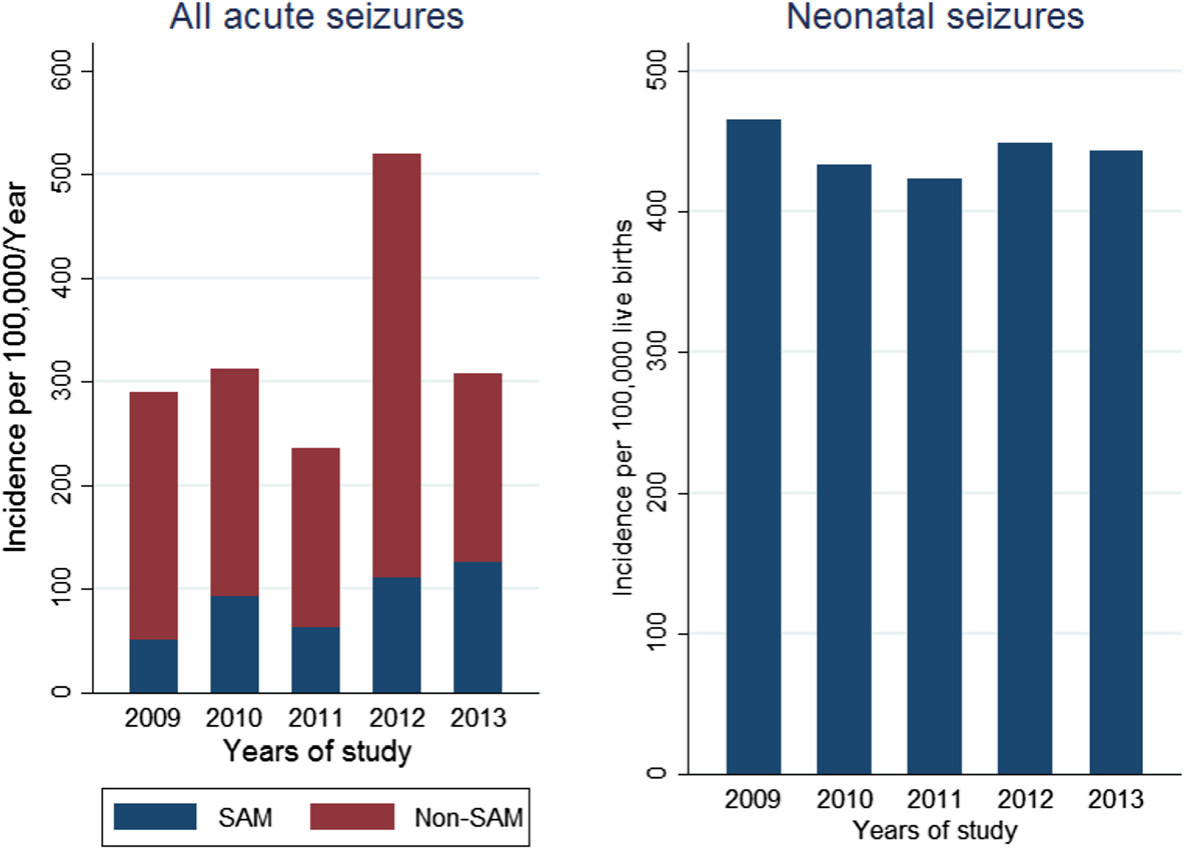
Incidence of admissions with acute seizures by year. Over the 5-year period, the incidence significantly increased for seizures-attributable to malaria (SAM), while non-SAM and neonatal seizures remained stable

**Fig. 3:**
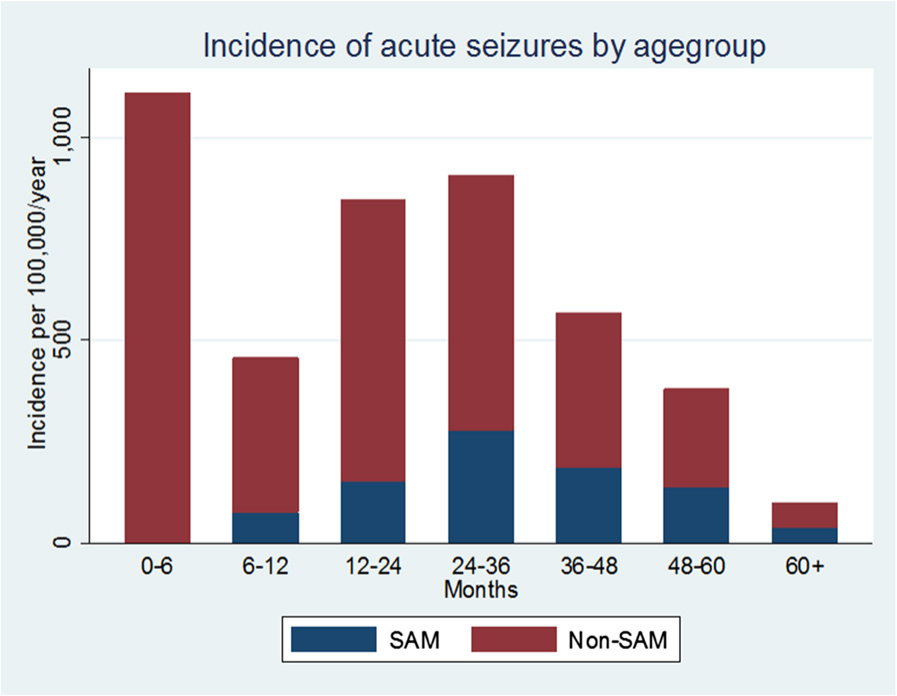
Incidence of admissions with acute seizures by age group. Malarial seizures were uncommon before 6 months age. The incidence for all seizures appeared increased up to 36 months then declined

The overall incidence of admissions with acute seizures was 312 per 100,000/year (95 % CI, 295–329), being 170 per 100,000/year (95 % CI, 157–182) in males. The overall incidence of acute seizures significantly increased with years of admission over the study period (incidence rate ratio (IRR) = 1.13; *p* < 0.001) (Fig. 2). Similarly, the overall incidence over the study period appeared to decrease with age (IRR = 0.651; *p* < 0.001) (Fig. 3). The incidence of all acute seizures appeared to decrease with distance from the hospital and was 257 per 100,000/year (95 % CI, 242–272) for those living within 5 kilometres from KCH, and 55 per 100,000/year (95 % CI, 48–62) for those living ≥5 kilometres from the hospital. The incidence of acute seizures among the neonates was 443 per 100,000 live births (95 % CI, 383–512), and remained stable over the five year period (IRR = 0.99 (95 % CI, 0.90-1.10); *p* = 0.905).

The incidence of complex seizures was 116 per 100,000/year (95 % CI, 106–127), and that for simple seizures was 196 per 100,000/year (95 % CI, 183–210). The incidence of complex seizures increased over the period (IRR = 2.07 (95 % CI, 1.90-2.26; *p* < 0.001)). The incidence for phenotypes of complex seizures was 40 per 100,000/year (95 % CI, 35–47) for prolonged seizures, 23 per 100,000/year (95 % CI, 19–28) for status epilepticus, 254 per 100,000/year (95 % CI, 240–270) for receptive seizures and 34 per 100,000/year (95 % CI, 29–40) for focal seizures.

### Incidence of seizures-attributable to malaria

The incidence of all seizures attributable to malaria (SAM) was 75 per 100,000/year (95 % CI, 67–83), and that for non-SAM was 237 per 100,000/year (95 % CI, 223–252). The incidence of SAM increased over the period (IRR = 1.22 (95 % CI, 1.13-1.31); *p* < 0.001) while that for non-SAM remained unchanged (IRR = 1.03 (95 % CI, 0.96-1.06); *p* = 0.569).

### Mortality in admissions with acute seizures

The overall case fatality proportion over the 5-year period was 6 % and remained stable throughout the study (*p* = 0.166). Death was more frequent in complex seizures than simple seizures (8 % vs.4 %; *p* < 0.001) and in neonatal seizures than seizures in those aged >28 days (10 % vs. 5 %; *p* = 0.005). In particular, 12 % of the deaths were in prolonged seizures and convulsive status epilepticus, 6 % were in repetitive seizures and 8 % in focal seizures. Mortality following acute seizures was reported in 20 % with malaria, 11 % with respiratory tract infections, 23 % with malnutrition and 12 % of encephalopathy with undetermined causes.

## Discussion

This study examined the effect of recent decline in malaria and increasing burden of neonatal admissions on the incidence of acute seizures, their proximate causes and phenotypes in children admitted to a rural hospital in Kenya. The burden of acute seizures in children remains high despite a reduction of malaria in the past decade. Indeed the overall incidence appeared to increase over the study period (2009–2013), as did the incidence of seizures attributable to malaria. The incidence of complex seizure increased the most (107 %). This suggests, in part, that causes of and risk factors for acute seizures are still prevalent in the community. Over 80 % of all incidence admissions resided less than 5 km from the KDH, implying hospital accessibility determines the admissions of acute seizures in this area. The most common causes of admissions with acute seizures were malaria and respiratory tract infections, but hypoglycemia, hypoxic-ischemic encephalopathy and neonatal sepsis were important among the neonates.

### Changes in incidence of acute seizures

The incidence of acute seizures in this study (312 per 100,000/year) is lower than that reported in a previous study (425 per 100,000/year) [1], which used data on admissions during a time when malaria was a common cause of morbidity and mortality in this area. Despite the recent decline in malaria, our present data reveals that the incidence has been increasing between 2009 and 2013, an important epidemiological finding which is not apparent if the present incidence was compared with earlier years when incidence of malaria was high.

This increase in incidence was particularly observed for SAM, being highest for the complex seizure phenotypes; supporting increasing burden for acute seizures post the malaria decline era. There are three explanations for the increasing incidence of seizures following the earlier decline. First and most plausible, a reduction in malaria may have resulted in less exposure, thereby reducing the acquisition of naturally acquired immunity to malaria, resulting in more severe illness, including seizures, that requires hospitalisation [5]. Secondly, this could be related to proportional increase in neonatal causes of seizures such as hypoglycemia, sepsis and hypoxic-ischemic encephalopathy compared to an earlier study [1]. Finally, the increasing incidence of acute seizures could be related to possible improvements in health care services not measured in this study, which would encourage admissions.

### Incidence of acute seizures

The incidence is grossly underestimated since only 28 % of it was from areas >5 kilometres. This suggests that poor infrastructure and transport costs may have resulted in some (if not most) seizures not being treated in hospital. In a recent study, only 56 % of persons with convulsive status epilepticus occurring with epilepsy were treated in KCH [17]. Other cultural reasons such as preference of traditional healers over biomedical practitioners may have contributed [18]. Despite the conservative incidence our estimates for prolonged and/or status epilepticus is higher than that reported in developed countries e.g. in London [19]. This may be explained by the high prevalence of infectious causes in this area e.g. malaria parasitemia, which was found in 36 % of all prolonged seizures; over 90 % of complex seizures in parasitemic children are caused by malaria [2]. Delays in management of acute seizures in peripheral clinics that may result in admission of seizures already refractory to treatment [14], adding to the high burden of prolonged seizures in this study.

### Causes of acute seizures

Malaria remains the most important cause of admission with seizures, with malaria parasitemia observed in over a third of admissions. This proportion however is slightly less than that reported in previous studies in this area (>50 %) [1, 14], suggesting a possibility of emergence of other important causes of acute seizures particularly those common in the neonatal period. Falciparum malaria may cause seizures by: (i) sequestration-induced brain damage that manifests as seizures [20]; (ii) down-regulation of GABA receptors thereby increasing susceptibility to seizures [21]; (iii) metabolic complications such as hyponatremia or hypoglycaemia and; (iv) induction of inflammatory molecules that lower seizure threshold [22]. Susceptibility to seizures may be determined by genetic polymorphisms [13].

Hypoglycemia, unknown encephalopathy and neonatal sepsis, were more common in the neonates in greater proportions than in a previous study [1], yet some of these can be easily prevented and managed. It is however, unclear if a proportion of encephalopathy of unknown origin reflects neurotropic viruses that were not investigated in this study [23].

Malaria was more common in complex seizures compared to simple seizures, suggesting a causal role for these complex phenotypes, as demonstrated in previous studies [2]. Additionally, other causes of seizures were significantly more frequent in complex seizures, perhaps because it represents a severe phenotype of seizures, as supported by its association with increased mortality in this study and neurocognitive impairments in previous studies [24, 25]. However, gastroenteritis appeared to be more frequent in simple seizures compared to complex seizures as would be expected.

### Changing phenotypes of acute seizures

Nearly half (46 %) of the admissions were complex seizures, a significant reduction from 70 % reported in previous studies in the same population [4, 13]. Since this phenotype is particularly attributable to malaria, the low frequency may be related to lower incidence of malaria during the study period compared to the early 2000s [5]. Complex seizures were associated with mean corpuscular volume, which may be a. marker of: (i) iron deficiency; (ii) red blood cell redistribution following sequestration into the deep capillary bends of the brain [26]; or (iii) presence of alpha thalassaemia [27].

### Mortality in acute seizures

The mortality from acute seizures was greater than in previous studies (6 % vs. 3 %) [1], probably suggesting a change in aetiology related to increase in neonatal seizures, in which mortality was highest (10 %). Additionally, case fatality was common (12 %) of those with encephalopathy of unknown origin which could be important in the neonatal period. Future studies should screen for viral aetiology of acute seizures, which may be associated with mortality but were not assessed in this study [28].

In terms of causes of acute seizures, mortality was highest in malaria (20 %) and malnutrition (20 %). Control of malaria and prevention of malnutrition would reduce just under half of mortalities associated with acute seizures.

### Strengths and limitations

These data were prospectively collected over a long time period with clinical and laboratory information documented in standardised proformas, thus findings are robust and reliable. Careful classification of acute seizures allowed objective relation of these phenotypes of seizures with causes, incidence and outcomes. These data may not represent the burden and situation of acute seizures in the community, since some seizures in this area are not treated at hospital. It was not possible to perform electroencephalography, owing to logistical constraints.

## Conclusions

The incidence of acute seizures is high in this area, and appears to be increasing during the period particularly for SAM, despite the reduction in the incidence of malaria. Malaria remains a common cause of seizures although in lesser proportions than previously documented while other etiologies in the neonatal period are emerging as important causes of seizures. The hospital-based incidence disproportionately represents only those living within a 5 kilometre radius and thus a true burden should be provided through community-based studies. Complex seizures remain a common phenotype, and should be addressed owing to their association with mortality. Mortality is still high particularly in neonatal seizures calling for plans to effectively prevent and manage seizures as well as control their associated causes and risk factors in the community.

## Abbreviations

CI: Confidence interval
ILAE: International League Against Epilepsy
IQR: Interquartile range
IRR: Incidence rate ratio
KCH: Kilifi County Hospital
SAM: Seizures-attributable to malaria
MUAC: Mid-upper arm circumference
WAZ: Weight-for-age z-scores

## References

[1] Idro R, Gwer S, Kahindi M, Gatakaa H, Kazungu T, Ndiritu M (2008). The incidence, aetiology and outcome of acute seizures in children admitted to a rural Kenyan district hospital. BMC Pediatr.

[2] Kariuki SM, Ikumi M, Ojal J, Sadarangani M, Idro R, Olotu A (2011). Acute seizures attributable to falciparum malaria in an endemic area on the Kenyan coast. Brain.

[3] Baram TZ, Shinnar S (2002). Febrile Seizures.

[4] Waruiru CM, Newton CR, Forster D, New L, Winstanley P, Mwangi I (1996). Epileptic seizures and malaria in Kenyan children. Trans R Soc Trop Med Hyg.

[5] O’Meara WP, Bejon P, Mwangi TW, Okiro EA, Peshu N, Snow RW (2008). Effect of a fall in malaria transmission on morbidity and mortality in Kilifi, Kenya. Lancet.

[6] Eckhoff PA (2012). Malaria parasite diversity and transmission intensity affect development of parasitological immunity in a mathematical model. Mal J.

[7] Mwaniki MK, Gatakaa HW, Mturi FN, Chesaro CR, Chuma JM, Peshu NM (2010). An increase in the burden of neonatal admissions to a rural district hospital in Kenya over 19 years. BMC Public Health.

[8] Njuguna P, Newton C (2004). Management of severe falciparum malaria. J Postgrad Med.

[9] Ogutu BR, Newton CR (2004). Management of seizures in children with falciparum malaria. Trop Doct.

[10] Beghi E, Carpio A, Forsgren L, Hesdorffer DC, Malmgren K, Sander JW (2010). Recommendation for a definition of acute symptomatic seizure. Epilepsia.

[11] Fisher RS, Acevedo C, Arzimanoglou A, Bogacz A, Cross JH, Elger CE (2014). ILAE Official Report: A practical clinical definition of epilepsy. Epilepsia.

[12] ILAE (1989). Proposal for revised classification of epilepsies and epileptic syndromes. Commission on Classification and Terminology of the International League Against Epilepsy. Epilepsia.

[13] Kariuki SM, Rockett K, Clark TG, Reyburn H, Agbenyega T, Taylor TE (2013). The genetic risk of acute seizures in African children with falciparum malaria. Epilepsia.

[14] Sadarangani M, Seaton C, Scott JA, Ogutu B, Edwards T, Prins A (2008). Incidence and outcome of convulsive status epilepticus in Kenyan children: a cohort study. Lancet Neurol.

[15] Berkley JA, Mwangi I, Ngetsa CJ, Mwarumba S, Lowe BS, Marsh K (2001). Diagnosis of acute bacterial meningitis in children at a district hospital in sub-Saharan Africa. Lancet.

[16] Bejon P, Berkley JA, Mwangi T, Ogada E, Mwangi I, Maitland K (2007). Defining childhood severe falciparum malaria for intervention studies. PLoS Med.

[17] Kariuki SM, Kakooza-Mwesige A, Wagner RG, Chengo E, White S, Kamuyu G (2015). Prevalence and factors associated with convulsive status epilepticus in Africans with epilepsy. Neurology.

[18] Kendall-Taylor N, Kathomi C, Rimba K, Newton CR (2008). Traditional healers and epilepsy treatment on the Kenyan coast. Epilepsia.

[19] Chin RF, Neville BG, Peckham C, Bedford H, Wade A, Scott RC (2006). Incidence, cause, and short-term outcome of convulsive status epilepticus in childhood: prospective population-based study. Lancet.

[20] Carter JA, Neville BGR, White S, Ross AJ, Otieno G, Mturi N (2004). Increased prevalence of epilepsy associated with severe falciparum malaria in children. Epilepsia.

[21] Ikumi ML, Muchohi SN, Ohuma EO, Kokwaro GO, Newton CR (2008). Response to diazepam in children with malaria-induced seizures. Epilepsy Res.

[22] Idro R, Jenkins NE, Newton CR (2005). Pathogenesis, clinical features, and neurological outcome of cerebral malaria. Lancet Neurol.

[23] D’Acremont V, Kilowoko M, Kyungu E, Philipina S, Sangu W, Kahama-Maro J (2014). Beyond malaria--causes of fever in outpatient Tanzanian children. N Engl J Med.

[24] Carter JA, Mung’ala-Odera V, Neville BGR, Murira G, Mturi N, Musumba C (2005). Persistent neurocognitive impairments associated with severe falciparum malaria in Kenyan children. J Neurol Neurosurg Psychiatry.

[25] Kariuki SM, Abubakar A, Newton CR, Kihara M (2014). Impairment of executive function in Kenyan children exposed to severe falciparum malaria with neurological involvement. Malaria J.

[26] Safeukui I, Buffet PA, Deplaine G, Perrot S, Brousse V, Ndour A (2012). Quantitative assessment of sensing and sequestration of spherocytic erythrocytes by the human spleen. Blood.

[27] Williams TN, Wambua S, Uyoga S, Macharia A, Mwacharo JK, Newton CRJC (2005). Both heterozygous and homozygous α + thalassemias protect against severe and fatal *Plasmodium falciparum* malaria on the coast of Kenya. Blood.

[28] Mallewa M, Vallely P, Faragher B, Banda D, Klapper P, Mukaka M (2013). Viral CNS infections in children from a malaria-endemic area of Malawi: a prospective cohort study. Lancet Glob Health.

